# A Mixture Fraction Approach to Predict Polymer Burning

**DOI:** 10.3390/polym16233313

**Published:** 2024-11-27

**Authors:** Artem Shaklein, Alexander Karpov, Stanislav Trubachev, Gabriela Morar, Nikita Balobanov, Ekaterina Mitriukova

**Affiliations:** 1 Udmurt Federal Research Center, Russian Academy of Science, Ural Branch, T. Baramzinoi, 34, 426067 Izhevsk, Russia; karpov@udman.ru (A.K.); morarga19@gmail.com (G.M.); nikitabalobanov225@gmail.com (N.B.); 2 Institute of Chemical Kinetics and Combustion, Russian Academy of Science, Siberian Branch, Institutskaya 3, 630090 Novosibirsk, Russia; satrubachev@gmail.com; 3 Mechanical Engineering Faculty, Rocket Engineering Department, Izhevsk State Technical University, Studencheskaya 7, 426069 Izhevsk, Russia; mit_e_a@mail.ru

**Keywords:** combustion, polymer, mixture fraction, kinetic mechanism, numerical simulation

## Abstract

A mixture fraction approach was applied to predict the combustion behavior of polymeric materials. In comparison to the combustion of gaseous mixtures, the presence of solid fuels complicates the description of the combustion. Accurate predictions of burning characteristics can only be achieved through the proper resolution of heat and mass transfer between the gas-phase flame and the solid fuel. We focused on a model case of flame spread over a solid fuel surface. Polymethyl methacrylate (PMMA) was selected as a polymeric material. An approach was proposed to account for heat loss from the gas phase to the solid material through calculations of counterflow diffusion flames with the flame positioned closely to the fuel supply. A combination of these solutions was applied to restore temperature and species mass fractions from tabulated chemistry. An analysis of the numerical results from previous studies on flame spread over PMMA, based on one-step combustion reaction and calculating the chemical source term at each time step, demonstrated a monotonic distribution of the mixture fraction in the flame region between the fuel and oxidizer streams. The shape of the flame tip was satisfactorily resolved using the proposed approach that employs a skeletal chemical mechanism for gas-phase combustion consisting of 29 species and 33 reactions. However, the heat flux from the flame to the solid fuel was overpredicted, resulting in higher flame spread rates compared to experimental data and previous calculations. Preliminary results show a promising opportunity for the mixture fraction approach to describe the combustion behavior of polymers. An analysis showed that oversimplifying the heat transfer process in the flame tip area is a main source of prediction inaccuracies. Multidimensional heat transfer has to be properly incorporated into a tabulated chemistry approach. Several potential directions for future work have been outlined.

## 1. Introduction

The combustion of solid fuels is a widespread phenomenon encountered in both nature (wildfires) and industry (heat engines, incineration factories). This sophisticated nature arises from the interaction of various factors, including the reacting gas flow, heat and mass transfer between the gas-phase flame and the solid combustible material, and the pyrolysis of the solid fuel.

The up-to-date level of combustion theory enables us to reduce pollutant formation, study material flammability, predict fire dynamics and flame spread, and enhance the stability of controlled combustion. Still, there are many ways to improve the accuracy and speed of calculations based on the theory. One method is discussed below. If gas is reactive, nonlinearity emerges from reaction rates, which are generally expressed in Arrhenius form with temperature under exponent. An expression for a reaction rate is introduced in species mass fraction and energy conservation equations as source terms. Generally, to properly resolve combustion processes, skeletal or detailed chemical kinetics mechanisms are often employed. These mechanisms consist of 10, 100, 1000, and more reactions, most of which have exponential dependence on temperature. Also, detailed chemical mechanisms often produce a stiff system of equations due to the presence of reactions with different time scales.

Many robust and fast methods for stiff systems have been developed and employed for the numerical integration of chemical source terms. Nevertheless, most of the computational time required for predicting the behavior of chemically reacting flows using detailed kinetic mechanisms is still spent on the calculation of a chemical source term.

Various approaches based on some assumptions have been introduced to simplify the nonlinear behavior of mathematical models for combustion and speed up calculations. One of them is a mixture fraction approach [1]. This was further developed into the flamelet approach by Peters [2], which became widespread to solve complex combustion problems.

**Figure 1:**
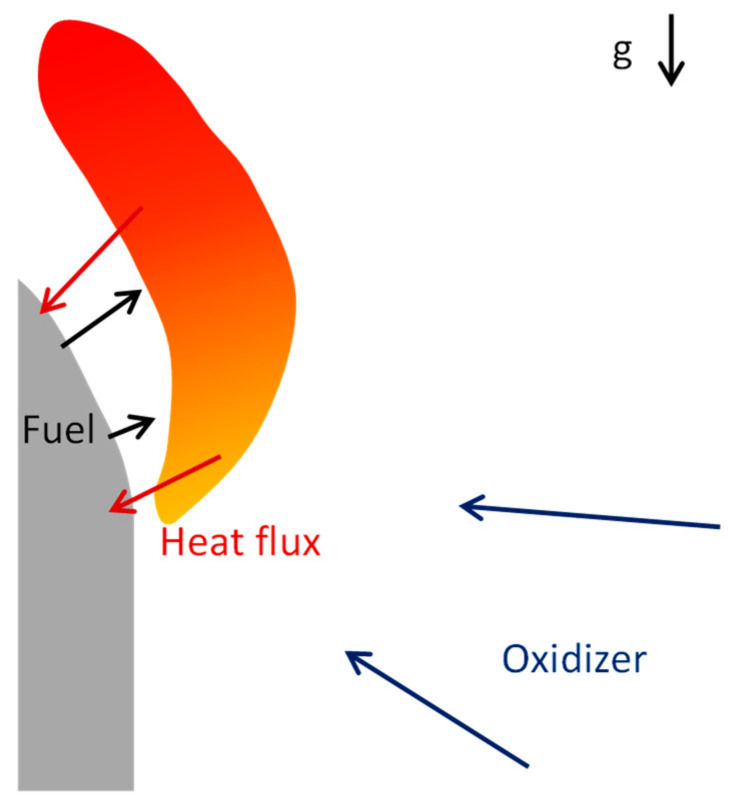
Scheme of a flame spread over solid combustible.

Modeling combustion of solid fuels in contrast to gas mixtures requires consideration of a heterogeneous system consisting of solid combustible material and reacting flow in the gas phase (Figure 1). Each point of a burning surface of a solid fuel provides a source of combustible gas with unique temperature and mass flow rate. Only accurate resolution of heat and mass transfer will provide adequate prediction of the burning behavior of solid fuel. Thus, existing models based on solving chemical kinetics at a preprocessing step could not be directly applied to the combustion of solid fuels and should be modified to take into account such effects.

Several studies focus on adapting a flamelet approach for the combustion of solid fuels. An applicability of a flamelet model (flamelet-generated manifolds model, FGM) for predicting the ignition and combustion of pulverized solid fuel has been tested using a laminar flat flame burner [3]. A gas-phase mechanism of coal and biomass combustion consisted of 68 species and 906 reactions. Chemical kinetics has been solved by a flamelet approach. Two mixture fractions were introduced to address the oxidation of methane and the volatile products of pulverized solid fuel pyrolysis. The study considered the heating of solid phase, and enthalpy was included as an additional dimension in the flamelet equations to account for non-adiabatic conditions. Some considerations of the flamelet application to solid fuel combustion are presented in [4]. Budzinski et al. [4] showed that specific areas are presented in the vicinity of the burning surface, which cannot be resolved by the flamelet approach (the FGM model was used). One such zone, located at the flame tip, is characterized by large multidirectional heat losses from gas to solid material.

Many skeletal and detailed chemical mechanisms have been developed, which can be applied to predict the combustion of pyrolysis products of polymers, such as methylmetacrylate [5,6], formaldehyde [7,8,9,10,11,12], styrene [12,13,14], and ethylene [15,16]. Often such mechanisms are used in simplified spatial configurations (mostly one-dimensional) due to high computational requirements of calculation of a chemical source term (polymethylmetacrylate [17], polyoxymethylene [18], polystyrene [18]). If more complex geometries are considered, basic chemical mechanisms (consisting of one or several reactions) are employed to predict combustion behavior of solid fuels, such as flame spread over combustibles [19,20], incineration of solid waste [21,22,23], burning of solid propellants [24,25], and forest fire spread [26]. Generally, these simplified mechanisms allow one to reasonably estimate global heat release of combustion but are unable to resolve local effects. For example, the two-step MMA combustion mechanism in comparison with the one-step macro-reaction gives noticeably better agreement between predictions and measurements on the thermal structure of flame spreading over PMMA surface [27].

Thus, in the current paper an attempt to apply a mixture fraction approach for combustion of solid fuels to increase usage of detailed combustion mechanisms is presented, and some preliminary findings are reported, which can suggest several directions for further development of a proposed methodology. A model case is examined here to study solid fuel combustion, specifically with a focus on the flame spread over the solid fuel surface. This configuration combines several useful features, such as two-dimensional formulation of conservation equations, dynamic behavior, and a steady spreading regime. Mostly, combustion occurs in a turbulent regime and includes a mix of diffusive and premixed flows. However, here, as a first step, we start from a laminar diffusion combustion model to simplify the formulation of the proposed methodology. This assumption eliminates the complex interaction between the flame and eddies, mitigating the impact of strong non-equilibrium effects associated with long chemical time scales. This allows one to focus primarily on calculating the chemical source term. This assumption is valid since flame spread over solid combustibles is controlled by processes that occur at the flame front, which is characterized by a laminar diffusion combustion regime. Later, this approach can be extended to include turbulent flames. Also, we have used several additional simplifications in order to focus on a general idea. Radiative heat transfer generally plays a minor but not negligible role; however, it was not considered here for a chosen specific flame spread configuration [28]. A flow regime was considered incompressible.

The formulation of reactive scalars, including equal diffusion coefficients, is applied here for simplification. An approach to include differential diffusion effects was proposed in [29] and can be used later as well. The Soret and Dufour effects were also not taken into account here.

We focus in our study on polymeric materials, which are highly presented nowadays in various fields, such as fuel cells [30], solid propellants [31], and electronic devices [32]. Polymer polymethylmethacrylate (PMMA, (C_5_H_8_O_2_)_n_)) was chosen as solid fuel for several reasons. PMMA almost fully thermally decomposes on its monomer, methylmethacrylate (MMA) [33]. PMMA was a model fuel for studying combustion, theoretically and experimentally, for many decades, and its physical and chemical properties have been thoroughly validated. Also, several chemical kinetic mechanisms of MMA combustion have already been proposed in the literature [5,6].

## 2. Mathematical Model

The mathematical formulation for the prediction of the combustion behavior of polymers is based on conservation equations and allows for the prediction of the integral (flame spread rate, fuel mass loss rate) and local (thermal and chemical structure of flame) characteristics. This model allows the accurate resolution of heat and mass transfer between the gas-phase flame and solid combustible material. It was thoroughly tested and validated on the combustion of various polymers [17,19,20]. The mathematical model of the gas phase takes into account multicomponent gas flow and combustion.

(1) $$\frac{\partial \rho }{\partial t}+\frac{\partial \rho u_{i}}{\partial x_{i}}=0,$$

(2) $$\rho \frac{\partial u_{i}}{\partial t}+\rho u_{j}\frac{\partial u_{i}}{\partial x_{j}}=-\frac{\partial p}{\partial x_{i}}+\frac{\partial }{\partial x_{j}}\mu \frac{\partial u_{i}}{\partial x_{j}}+\left(\rho -\rho_{a}\right)g_{i}$$

(3) $$\rho \frac{\partial Y_{k}}{\partial t}+\rho u_{i}\frac{\partial Y_{k}}{\partial x_{i}}=\frac{\partial }{\partial x_{i}}\rho D\frac{\partial Y_{k}}{\partial x_{i}}+{\dot{\omega }}_{k},$$

(4) $$\rho c_{p}\frac{\partial T}{\partial t}+\rho u_{i}c_{p}\frac{\partial T}{\partial x_{i}}=\frac{\partial }{\partial x_{i}}\lambda \frac{\partial T}{\partial x_{i}}-\sum_{k=1}h_{k}{\dot{\omega }}_{k},$$

(5) $$p=\frac{\rho R_{0}T}{M},$$

where ρ is density, u—velocity, t—time, x—coordinate, p—pressure, μ—viscosity, g—gravitational acceleration, $Y_{k}$—mass fraction of species k, D—species mass diffusivity, $\dot{\omega }$—mass production rate, T—temperature, $c_{p}$—specific heat capacity at constant pressure, λ—thermal conductivity, h—enthalpy, $R_{0}$—universal gas constant, M—molecular weight, and subscript a stands for ambient.

The mathematical model of solid fuel is presented next [17,19,20]. It takes into account heat transfer and pyrolysis of solid material. A mass flow rate of gaseous pyrolysates is calculated based on an integral of a pyrolysis reaction rate. Such formulation allows one to accurately resolve the heating of solid material by flame and the release of combustible gas.

The thermal state of a solid combustible is described by the energy equation

(6) $$\rho_{s}c_{s}\frac{\partial T_{s}}{\partial t}=\frac{\partial }{\partial x_{i}}\lambda_{s}\frac{\partial T_{s}}{\partial x_{i}}+\rho_{s}W_{s}Q_{s},$$

where W is a reaction rate, c—specific heat capacity, Q—heat effect of a reaction, and subscript s stands for solid.

Solid fuel under external heat flux from the flame decomposes on combustible products. Here, a one-step pyrolysis reaction is considered:

(7) Solid fuel→Gaseous fuel,

which rate is expressed in the Arrhenius form

(8) $$W_{s}={\left(1-\alpha_{s}\right)}^{n_{s}}k_{s}\exp\left(-E_{s}/\left(R_{0}T_{s}\right)\right),$$

where α is a reaction progress, n—reaction order, k—pre-exponential factor, and E—activation energy.

Pyrolysis reaction progress is defined as

(9) $$\frac{d\alpha_{s}}{dt}=W_{s}.$$

Gaseous combustible products of a pyrolysis reaction flow to a burning surface and go to the gas phase with mass flux calculated as

(10) $${\dot{m}}_{s}(y)=\rho_{s}\int_{-L_{s}}^{0}W_{s}\mathrm{dx},$$

where $\dot{m}$ is a mass flow rate; $L_{s}$—thickness of solid fuel sample.

According to general procedure [34], a steady state mixture fraction approach is defined in the following way

(11) $$\rho \frac{\partial Z}{\partial t}+\rho u_{i}\frac{\partial Z}{\partial x_{i}}=\frac{\partial }{\partial x_{i}}\rho D\frac{\partial Z}{\partial x_{i}},$$

(12) $$\frac{1}{2}\rho \chi \frac{\partial^{2}Y_{k}}{\partial Z^{2}}+{\dot{\omega }}_{k}=0,$$

(13) $$\frac{1}{2}\rho \chi \frac{\partial^{2}T}{\partial Z^{2}}-\frac{1}{c_{p}}\sum_{k=1}^{K}h_{k}{\dot{\omega }}_{k}=0,$$

where Z is mixture fraction and $\chi =2D{\left(\partial Z/\partial x_{i}\right)}^{2}$ is scalar dissipation rate.

We have used a counterflow flame approach to generate solutions to Equations (12) and (13) in a mixture fraction space. In contrast to pure gas-phase combustion, a solid source for gaseous fuel introduces additional complexities. Firstly, some of the heat generated during combustion is transferred to the solid fuel. Such heat losses of gas mixture energy are substantial in comparison, for example, to radiative heat losses, which can be neglected with reasonable accuracy for downward flame spread [28]. Therefore, the combustion process cannot be considered adiabatic, and heat losses must be accounted for using a chemistry tabulation procedure. Secondly, the entire burning surface generates gaseous combustible fuel, and the temperature of this surface can vary significantly. Thus, it is essential to consider the supply of volatile fuel at different temperatures.

An approach to resolve such issues is discussed next. One-dimensional steady equations of counterflow flame configuration are presented as follows: [35]

(14) $$\frac{d\rho u}{dx}+2\rho V=0,$$

(15) $$\rho u\frac{dV}{dx}+\rho V^{2}=-\Lambda +\frac{d}{dx}\mu \frac{dV}{dx},$$

(16) $$\rho uc_{p}\frac{dT}{dx}=\frac{d}{dx}\lambda \frac{dT}{dx}-\sum_{k}h_{k}{\dot{\omega }}_{k}-\sum_{k}c_{pk}j_{k}\frac{dT}{dx},$$

(17) $$\rho u\frac{dY_{k}}{dx}=-\frac{dj_{k}}{dx}+{\dot{\omega }}_{k},$$

(18) $$p=\frac{\rho R_{0}T}{M},$$

where u, v—axial and radial velocity components, V=v/r—dimensionless radial velocity, Λ—pressure eigenvalue, $j_{k}=j_{k}^{*}-Y_{k}\sum_{i}j_{i}^{*}$—species diffusion mass flux [35], $j_{k}^{*}=-\rho \left(M_{k}/M\right){D^{\prime }}_{km}\left(dX_{k}/dz\right)$, ${D^{\prime }}_{km}$—the mixture-averaged diffusion coefficient for species k, and $X_{k}$ is the mole fraction for species k.

The discussion on the evaluation of species mass diffusion using a mixture fraction approach [29,36] shows that the problem is quite complex. Here we calculated solutions of reactive scalars (temperature and species mass fractions) in a mixture fraction space, taking into account diffusion fluxes estimated individually for each species, Equation (17). We used the formulation of reactive scalars, Equations (11)–(13), arranged by using a single diffusion coefficient D, approximated as that of the mixture, to simplify our analysis.

The last term in energy Equation (16) is small compared to a source term and usually neglected (as was completed for Equation (4)) [2].

**Figure 2:**
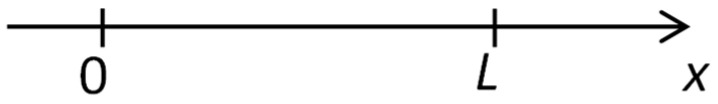
The computational domain of counterflow diffusion flame.

Corresponding boundary conditions are set in accordance with Figure 2.

(19) $$x=0:\;\rho u={\dot{m}}_{f},\;T=T_{f},\;j_{k}+\rho uY_{k}={\dot{m}}_{f}Y_{kf},$$

(20) $$x=L:\;\rho u=-{\dot{m}}_{o},\;T=T_{o},\;j_{k}+\rho uY_{k}=-{\dot{m}}_{o}Y_{ko},$$

where subscript f stands for a fuel supply; subscript o stands for an oxidizer supply.

To account for the heat losses from the gas phase to a solid material, we set the fol-lowing approach. A position of flame (maximum temperature) in counterflow configura-tion results from various parameters, such as mass rates of inflow streams. Usually, the flame is set in the middle of a computational domain, with nearly constant profiles of temperature and concentrations near the boundaries. In order to mimic the behavior of solid fuel combustion in a counterflow configuration, the flame has to be moved closer to the fuel supply. This operation is carried out by the modification of the fuel mass inflow rate. We observed that as the flame approaches the fuel inlet boundary with variations in the fuel mass inflow rate, the mixture fraction at that boundary decreases. So, in accord-ance to each value of the fuel flow rate we get a specific value of *Z* at the burning surface, *Z_w_*.

During the calculations of flame spread, values of Z are known at each iteration, and $Z_{w}$ are also known. Thus, we can consider Z to be the mix of various counterflow solutions providing heat flux from gas to solid phase. This Z field is the combination of solutions of various fuel mass inflow rates (and, therefore, $Z_{w}$). These solutions are combined in the following way. We propose, that Z is a linear combination of several solutions such as

(21) $$Z\left(x_{i},t\right)=\sum_{k}Z_{k}\left(x_{i},t\right),$$

where k varies from 1 to N, where N is the number of selected solutions.

This procedure can be considered similar to the mass conservation equations of mixture and species.

Substitution of (21) in (11) gives

(22) $$\rho \frac{\partial \sum_{k}Z_{k}}{\partial t}+\rho u_{i}\frac{\partial \sum_{k}Z_{k}}{\partial x_{i}}=\frac{\partial }{\partial x_{i}}\rho D\frac{\partial \sum_{k}Z_{k}}{\partial x_{i}},$$

We can split this equation to get a system of n equations in the following way

(23) $$\rho \frac{\partial Z_{k}}{\partial t}+\rho u_{i}\frac{\partial Z_{k}}{\partial x_{i}}=\frac{\partial }{\partial x_{i}}\rho D\frac{\partial Z_{k}}{\partial x_{i}},$$

We divide the whole range of available Z values, which is from 0 to 1, by N equal segments, $\zeta_{k}$. As a result, we get N values of $\zeta_{k}$, each set to a specific $Z_{k}$ function.

During the calculation procedure, we get $Z_{wj}$ at each boundary face j of the burning surface at each iteration from a boundary condition $-\rho D\left(\partial Z/\partial x\right)+\rho uZ={\dot{m}}_{s}Z_{s}$. $Z_{s}$ is 1 for a fuel stream according to a mixture fraction approach. Then we place each boundary value of $Z_{wj}$ between two nearby values of $\zeta_{k}$ and $\zeta_{k+1}$. By applying linear interpolation, we get corresponding weights, $\alpha_{k}$ and $1-\alpha_{k}$. At this boundary point j, all $Z_{kwj}$ values are zero except for those corresponding to $\zeta_{k}$ and $\zeta_{k+1}$. Further, $Z_{kwj}=\alpha_{k}Z_{wj}$, $Z_{\left(k+1\right)wj}=\alpha_{k+1}Z_{wj}$. After defining the boundary values, we can solve Equation (23) for each $Z_{k}$.

By applying this method, we are able to parameterize a solution by variation of heat flux. So, we can obtain the tabulated temperature as $T=f_{T}\left(Z_{k}\right)$.

In order to take into consideration the variable temperature of the burning surface, we parameterized solution, as was usually carried out for non-adiabatic flamelets. We can split Equation (4) by employing $T\left(x_{i},t\right)=T_{c}\left(x_{i},t\right)+T^{\prime }\left(x_{i},t\right)$, where subscript c stands for an energy part, including a chemical source term, and get

(24) $$\rho c_{p}\frac{\partial T_{c}}{\partial t}+\rho u_{i}c_{p}\frac{\partial T_{c}}{\partial x_{i}}=\frac{\partial }{\partial x_{i}}\lambda \frac{\partial T_{c}}{\partial x_{i}}-\sum_{k=1}h_{k}{\dot{\omega }}_{k},$$

(25) $$\rho c_{p}\frac{\partial T^{\prime }}{\partial t}+\rho u_{i}c_{p}\frac{\partial T^{\prime }}{\partial x_{i}}=\frac{\partial }{\partial x_{i}}\lambda \frac{\partial T^{\prime }}{\partial x_{i}},$$

Using enthalpy instead of temperature in this procedure would provide clearer and more accurate results. However, for the sake of simplifying calculations, we chose to use temperature as a first approximation.

Thus, the solution of (25) provides the value of an additional $T^{\prime }$ to be applied to function $T_{c}$ to evaluate T at any point. One-dimensional counterflow solutions are parameterized by varying temperature of a fuel inlet. So by this approach, we can treat the variable temperature of the burning surface as $T=f_{T}\left(Z_{k},T^{\prime }\right)$.

**Figure 3:**
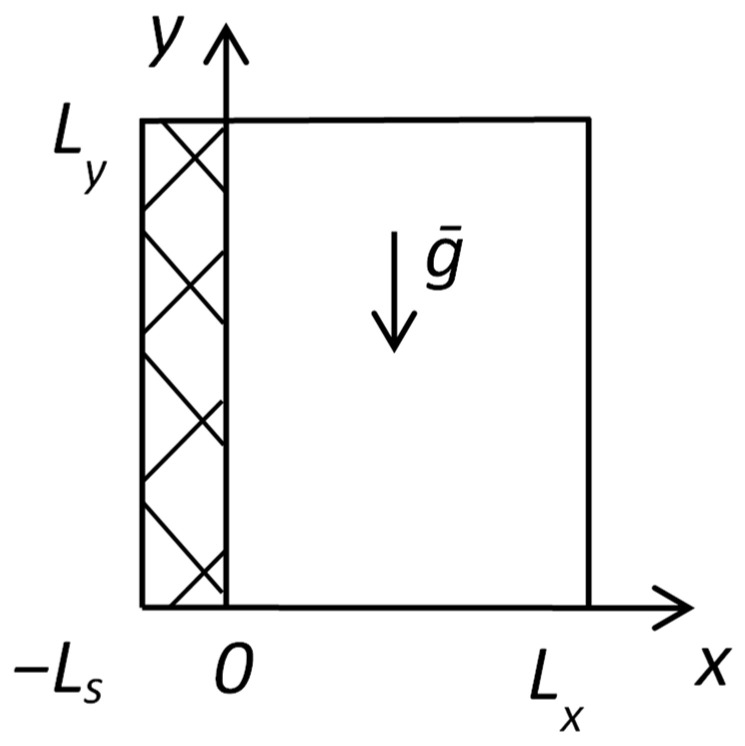
The computational domain of flame spread over solid fuel.

The following boundary conditions are set for the mathematical model describing flame spread over solid fuel according to Figure 3.

Gas phase:

Opening

(26) $$y=0,\;x=L_{x}:\;Z=0,\;Z_{k}=0,\;T^{\prime }=0,\;\partial u/\partial n=0,\;\partial v/\partial n=0;$$

Outflow

(27) $$y=L_{y}:\;\partial \phi /\partial y=0,\;\phi =\left\{u,v,Z,Z_{k},T^{\prime }\right\};$$

Solid phase:

(28) $$y=0,\;x=-L_{s},\;y=L_{y}:\;\partial T_{s}/\partial n=0;$$

Burning surface:

(29) $$\begin{array}{ll} x=0,\;0<y<L_{y}:\; & \rho u={\dot{m}}_{s},\;T^{\prime }=T_{s}-T_{c},\;T_{c}=T_{a},\\ & -\lambda \left(\partial T/\partial x\right)+\rho uc_{p}T=-\lambda_{s}\left(\partial T_{s}/\partial x\right)+{\dot{m}}_{s}c_{s}T_{s},\\ & -\rho D\left(\partial Z/\partial x\right)+\rho uZ={\dot{m}}_{s}Z_{s},\;Z_{s}=1,\\ & -\rho D\left(\partial Z_{k}/\partial x\right)+\rho uZ_{k}={\dot{m}}_{s}Z_{ks}; \end{array}$$

Initial conditions:

(30) $$t=0:\;u=0,\;v=0,\;Z=0,\;Z_{k}=0,\;T^{\prime }=0,\;\alpha =0,\;T_{s}=T_{a}.$$

### 2.1. Mixture Fraction Definition

A definition of mixture fraction required careful analysis. General ways to calculate mixture fraction are (according to [29,34]) based on mass fractions of fuel and oxidizer and elements

(31) $$Z=\frac{\nu Y_{F}-Y_{O_{2}}+Y_{O_{2},2}}{\nu Y_{F,1}+Y_{O_{2},2}},$$

and

(32) $$Z=\frac{Z_{C}/\left(n_{C}M_{C}\right)+Z_{H}/\left(n_{H}M_{H}\right)+2\left(Y_{O_{2},2}-Z_{O}\right)/\left({\nu^{\prime }}_{O_{2}}M_{O_{2}}\right)}{Z_{C,1}/\left(n_{C}M_{C}\right)+Z_{H,1}/\left(n_{H}M_{H}\right)+2Y_{O_{2},2}/\left({\nu^{\prime }}_{O_{2}}M_{O_{2}}\right)},$$

where $\nu =\left({\nu^{\prime }}_{O_{2}}M_{O_{2}}\right)/\left({\nu^{\prime }}_{F}M_{F}\right)$ is the mass oxidizer-to-fuel stoichiometric ratio, M is molar mass, $Z_{k}$ is mass fraction of element k, $n_{k}$ is the number of elements k in specie (fuel or oxidizer), indexes ‘1’ and ‘2’ correspond to fuel and oxidizer supply, $\nu^{\prime }$ is a stoichiometric coefficient defined for a macro-reaction ${\nu^{\prime }}_{F}F+{\nu^{\prime }}_{O_{2}}O_{2}\to {\nu^{\prime \prime }}_{P}P$, where F is a fuel, and P is products.

This paper focuses on the combustion of methyl methacrylate (MMA, C_5_H_8_O_2_), an ester that contains oxygen elements. Oxygen-containing molecules found in combustion products cannot be directly attributed to their source (the fuel or the oxidizer mixture), thus Equation (32) cannot be used in the present form. Therefore, we need to establish a broader definition of the mixture fraction, which would take into account oxygen atoms in the fuel. Considering the general form of fuel $C_{n_{C}}H_{n_{H}}O_{n_{O}}$ and oxidizer $O_{2}$, we get

(33) $$\frac{Z_{C,F}}{n_{C}M_{C}}=\frac{Z_{H,F}}{n_{H}M_{H}}=\frac{Z_{O,F}}{n_{O}M_{O}}=\frac{Y_{F,u}}{M_{F}},\;Z_{O,O}=Y_{O_{2},u},$$

where $Z_{ki}$—mass fraction of k-th element from i-th species; subscript u stands for initial (unburnt) mixture.

At stoichiometry, the following relation holds

(34) $${\frac{Y_{O_{2},u}}{Y_{F,u}}|}_{st}=\frac{{\nu^{\prime }}_{O_{2}}M_{O_{2}}}{{\nu^{\prime }}_{F}M_{F}}=\nu ,$$

or

(35) $$\frac{Y_{F,u}}{{\nu^{\prime }}_{F}M_{F}}=\frac{Y_{O_{2},u}}{{\nu^{\prime }}_{O_{2}}M_{O_{2}}}.$$

A function $\beta \left(Z_{C},Z_{H},Z_{O}\right)$ is introduced, which is defined as the difference between terms in Equation (35)

(36) $$\beta =\frac{Y_{F,u}}{{\nu^{\prime }}_{F}M_{F}}-\frac{Y_{O_{2},u}}{{\nu^{\prime }}_{O_{2}}M_{O_{2}}}.$$

This function becomes zero at stoichiometry.

Substitution of (33) in (36) gives

(37) $$\beta =\frac{Z_{C,F}}{{\nu^{\prime }}_{F}n_{C}M_{C}}-\frac{Z_{O,O}}{{\nu^{\prime }}_{O_{2}}M_{O_{2}}}.$$

To eliminate the oxygen provided by the oxidizer, we need to adjust Equation (37) accordingly. Taking into account that the oxygen element in the combustion area originates solely from fuel or oxidizer supplies, $Z_{O}=Z_{O,F}+Z_{O,O}$ we get

(38) $$\begin{array}{l} \beta =\frac{Z_{C,F}}{{\nu^{\prime }}_{F}n_{C}M_{C}}-\frac{Z_{O,O}}{{\nu^{\prime }}_{O_{2}}M_{O_{2}}}=\frac{Z_{C,F}}{{\nu^{\prime }}_{F}n_{C}M_{C}}-\frac{Z_{O}-Z_{O,F}}{{\nu^{\prime }}_{O_{2}}M_{O_{2}}}=\\ =\frac{Z_{C,F}}{{\nu^{\prime }}_{F}n_{C}M_{C}}+\frac{Z_{O,F}}{{\nu^{\prime }}_{O_{2}}M_{O_{2}}}-\frac{Z_{O}}{{\nu^{\prime }}_{O_{2}}M_{O_{2}}}. \end{array}$$

Substitution of $Z_{O,F}$ by $Z_{H,F}$ according to Equation (33) gives

(39) $$\beta =\frac{Z_{C,F}}{{\nu^{\prime }}_{F}n_{C}M_{C}}+\frac{Z_{H,F}}{{\nu^{\prime }}_{O_{2}}M_{O_{2}}}\frac{n_{O}M_{O}}{n_{H}M_{H}}-\frac{Z_{O}}{{\nu^{\prime }}_{O_{2}}M_{O_{2}}}.$$

Since carbon and hydrogen elements come to domain only from fuel supply, then $Z_{C}=Z_{C,F}$ and $Z_{H}=Z_{H,F}$; thus, Equation (39) becomes

(40) $$\beta =\frac{Z_{C}}{{\nu^{\prime }}_{F}n_{C}M_{C}}+\frac{Z_{H}}{{\nu^{\prime }}_{O_{2}}M_{O_{2}}}\frac{n_{O}M_{O}}{n_{H}M_{H}}-\frac{Z_{O}}{{\nu^{\prime }}_{O_{2}}M_{O_{2}}}.$$

Mixture fraction can be obtained by the normalization of β in the following way

(41) $$Z=\frac{\beta -\beta_{2}}{\beta_{1}-\beta_{2}},$$

where subscript ‘1’ stands for a fuel supply, subscript ‘2’—an oxidizer supply.

Substitution of (40) into (41) gives

(42) $$Z=\frac{\frac{Z_{C}}{{\nu^{\prime }}_{F}n_{C}M_{C}}+\frac{Z_{H}}{{\nu^{\prime }}_{O_{2}}M_{O_{2}}}\frac{n_{O}M_{O}}{n_{H}M_{H}}-\frac{Z_{O}}{{\nu^{\prime }}_{O_{2}}M_{O_{2}}}+\left(\frac{Y_{O_{2},u}}{{\nu^{\prime }}_{O_{2}}M_{O_{2}}}\right)}{\left(\frac{Z_{C,1}}{{\nu^{\prime }}_{F}n_{C}M_{C}}+\frac{Z_{H,1}}{{\nu^{\prime }}_{O_{2}}M_{O_{2}}}\frac{n_{O}M_{O}}{n_{H}M_{H}}-\frac{Z_{O,1}}{{\nu^{\prime }}_{O_{2}}M_{O_{2}}}\right)+\left(\frac{Y_{O_{2},u}}{{\nu^{\prime }}_{O_{2}}M_{O_{2}}}\right)},$$

The formulation of mixture fraction presented in Equation (42) allows its application to the combustion of MMA in air.

### 2.2. Numerical Approach

A summary of a numerical approach is given below. The system of Equations (1), (2), (6), (9), (11), (23), and (25) is solved with the corresponding boundary conditions, Equations (26)–(30), in a modified version of OpenFOAM version 8 [37]. A general algorithm is discussed and tested elsewhere [19,20]. In the present study, due to the use of a tabulated chemistry approach, Equations (11), (23), and (25) are solved instead of Equations (3) and (4) at each iteration within a time step loop. At the next step, the temperature and species mass fractions are restored based on the tabulated data.

The set of one-dimensional steady Equations (14)–(17) representing counterflow diffusion flame with the corresponding boundary conditions, Equations (19) and (20), is solved in Cantera [35]. The solution procedure was recently tested on the combustion of polymers [17,18].

## 3. Results and Discussion

### 3.1. Preliminary Analysis

**Figure 4:**
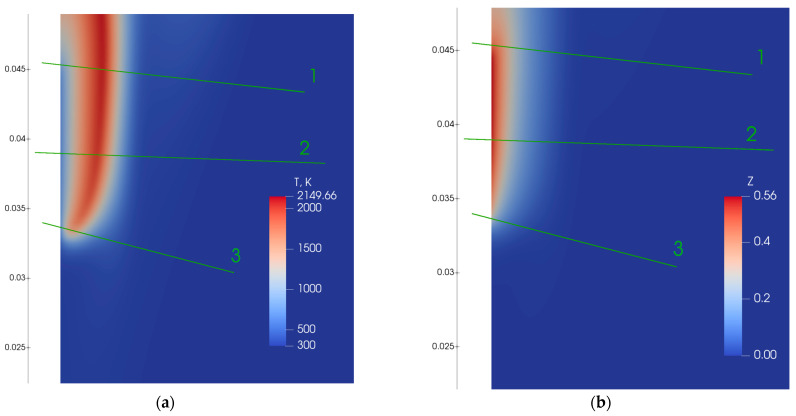
Distribution of predicted temperature (**a**) and mixture fraction (**b**).

**Figure 5:**
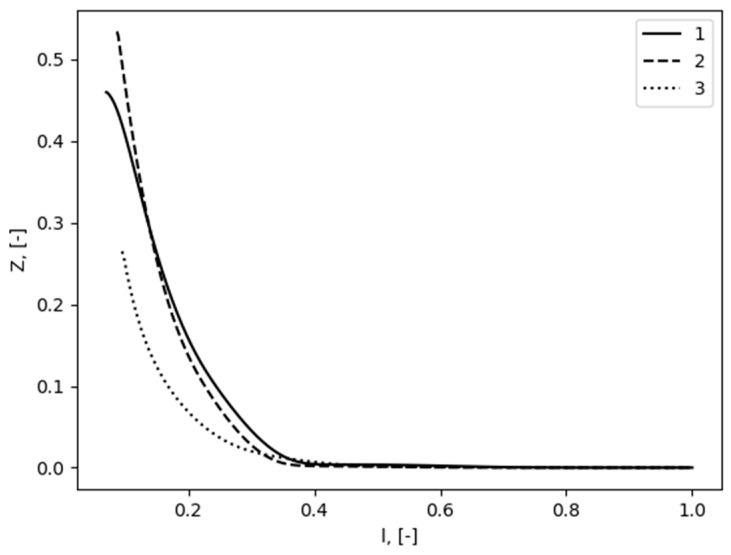
Distribution of mixture fraction along lines drawn in Figure 4; horizontal axis are attributed to the relative distance along each curve.

The next section presents the verification of the monotonous distribution of the mixture fraction for solid fuel combustion. We used data on of flame spread over PMMA calculated earlier, with the corresponding numerical formulation, input data, and results presented in [27]. In this model, we employed a one-step gas-phase reaction for combustion and calculated its rate at each time step. We used these results in the present study to analyze the distribution of mixture fraction in a flame zone. The question was—does mixture fraction vary monotonically between fuel and oxidizer inlets? Results are presented at Figure 4. The lines ‘1’, ‘2’, and ‘3’ were chosen somewhat arbitrarily to demonstrate a general trend of mixture fraction distribution in the flame zone, including the flame tip. We used Equation (42) to calculate mixture fraction values. Only fuel (MMA), oxidizer (O_2_), product (CO_2_ and H_2_O), and inert component (N_2_) were considered, but mass fractions of elements still can be calculated. Z monotonically varies from maximum at a burning surface to minimum in the environment. Mixture fraction profiles along lines ‘1’, ‘2’, and ‘3’ are shown in Figure 5, demonstrating the monotonic distribution of mixture fraction. This observation shows that a mixture fraction approach can be applied to predict the behavior of the flame spread over polymers.

### 3.2. Tabulating Chemistry

The dependence of temperature and species mass fractions on mixture fraction was calculated using the one-dimensional counterflow diffusion flame model presented above. The following input values were used. Computational domain length was L = 15.4 mm (Figure 2). A skeletal chemical mechanism for gas-phase combustion was used, consisting of 29 species and 33 reactions [5]. The boundary conditions parameters are as follows: ${\dot{m}}_{o}$ = 0.39 kg/(m^2^∙s), $Y_{O_{2},o}$ = 0.233, $Y_{N_{2},o}$ = 0.767, $T_{o}$ = 300 K, $Y_{MMA,f}$ = 1. The pressure is 1 atm. Values of temperatures and mass inflow rates of the fuel boundary are $T_{f}$ = 300, 400, 500, 650, 700, 750, 800 K, ${\dot{m}}_{f}$ = 0.001, 0.004, 0.0045, 0.005, 0.007, 0.012, 0.02, 0.1 kg/(m^2^∙s). Depending on the case, the non-uniform grid has 50–1000 points and a minimum size of 3 × 10^−4^–5 × 10^−9^ m.

**Figure 6:**
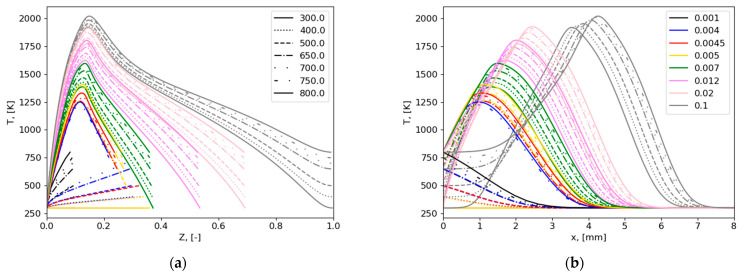
Temperature profiles over mixture fraction (**a**) and physical coordinate (**b**); legend shows values of fuel temperatures (**a**) K and mass inflow rates (**b**) kg/(m^2^∙s); note that Z = 1 and x = 0 corresponds to the fuel stream.

The distribution of temperature in the computational domain, in both mixture fraction space and physical space, is presented in Figure 6.

Tabulation was performed by dividing the mixture fraction space into 40 points.

### 3.3. Flame Spread over PMMA

The behavior of the flame spread over the surface of the 5 mm thick PMMA sample was predicted based on the approach presented above. Geometrical parameters of the computational domains are as follows (Figure 3): $L_{x}$ = 25 mm, $L_{y}$ = 100 mm, $L_{s}$ = 2.5 mm (we consider only half of the sample due to the symmetry of the configuration). The physical properties of PMMA are [38]: $\rho_{s}$ = 1190 kg/m^3^, $\lambda_{s}$ = 0.188 W/(m∙K), $c_{s}$ = 1465 J/(kg∙K). The kinetic parameters of the pyrolysis reaction are [27]: $k_{s}$ = 4.75∙10^12^ 1/s, $E_{s}$ = 177.6 kJ/mol, $n_{s}$ = 1.3 and $R_{0}$ = 8.314 kJ/(mol K). The heat effect of the pyrolysis reaction is $Q_{s}$ = −0.94 MJ/kg [38]. Ambient temperature is $T_{a}$ = 300 K. The gravity vector is represented as $g_{x}$ = 0, $g_{y}$ = ±9.81 m/s^2^ since a downward flame spread configuration was chosen. The two-dimensional formulation of the numerical model was used.

The gas-phase computational domain was split non-uniformly in the direction normal to the burning surface by 300 elements, with the minimal size at the burning surface of 5 × 10^−5^ m. In the direction parallel to the burning surface of the polymer, both gas-phase and solid domains were split by 180 uniform points for 0.09 m with the mesh size of 5 × 10^−4^ m and 10 non-uniform points to cover the remaining 0.01 m of the top part of the domains. In the solid sample normal to the burning surface, 70 uniform points were chosen with the mesh size of 3.57 × 10^−5^ m.

The flame was ignited by applying a volumetric heat source in the solid computational domain in the vicinity of the burning surface. After ignition, the heat source was turned off, allowing the flame to spread by itself without any external forcing.

**Figure 7:**
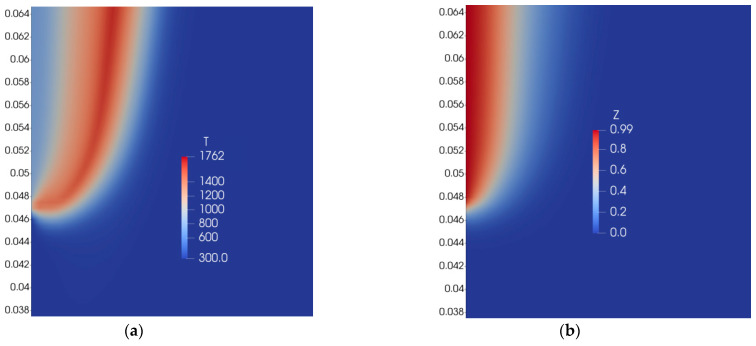
Distribution of temperature of gas phase (**a**) and mixture fraction (**b**).

The distribution of gas-phase temperature and mixture fraction is presented in Figure 7.

The valuable result obtained here is the flame tip shape (Figure 7), which is qualitatively close to the one predicted by the general approach with one-step combustion reaction (Figure 4). The general approach means calculation of a chemical source term inside the main time loop.

**Figure 8:**
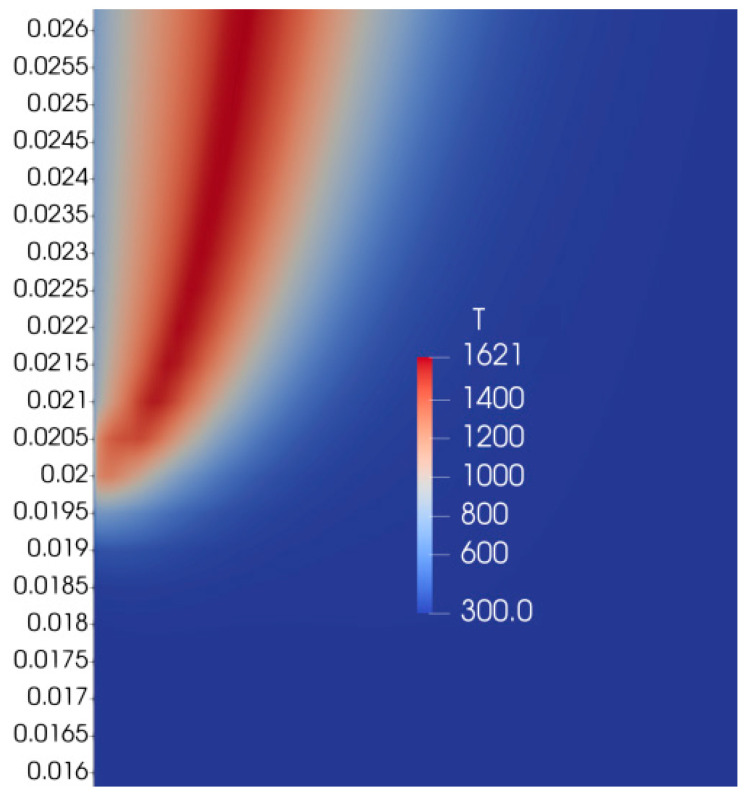
Distribution of gas-phase temperature.

If we apply a mixture fraction approach without splitting Z by $Z_{k}$ (Equation (23)), we observe that the flame tip widens (Figure 8), moves closer to the burning surface, which results in a higher flame spread rate compared to the case with combination of solutions of various fuel mass inflow rates.

Also, we tried various dependences of temperature on the scalar dissipation rate, but we were unable to even get the satisfactory shape of the flame tip.

**Figure 9:**
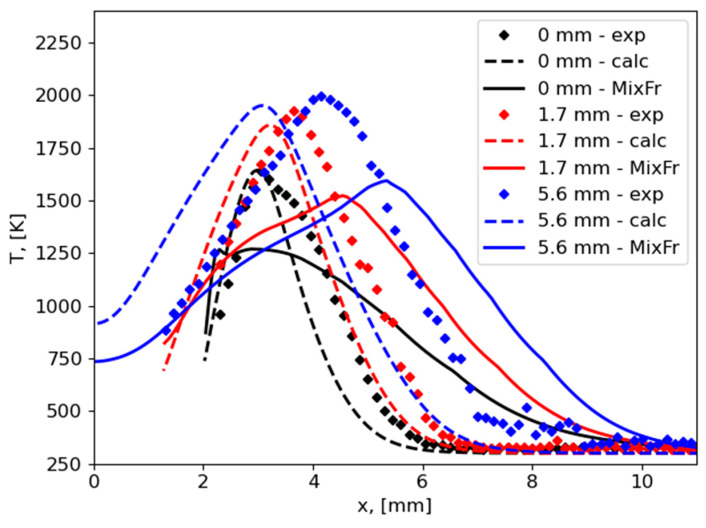
Temperature profiles along the direction normal to the burning surface, measurements (exp) [27], calculations by the one-step combustion reaction (calc) [27], predictions by the mixture fraction approach (MixFr); distances (0, 1.7, 5.6 mm) are from the flame front.

This preliminary result is still far from the accurate prediction of flame spread by a mixture fraction approach. Flame spread rate was almost 10 times higher than measured and predicted using the general approach with a one-step combustion reaction [27]. Also, values of Z in the vicinity of the burning surface are higher for a mixture fraction approach (Figure 7b) in comparison to the general approach (Figure 4b). Temperature profiles measured [27], calculated by the one-step combustion reaction [27], and predicted by the mixture fraction approach along the direction normal to the burning surface are presented in Figure 9. The agreement here is also not satisfactory.

We propose the following explanation of such differences. The heat transfer from flame to solid fuel is a controlling mechanism of flame spread. High flame spread rate implies the excess of heat supplied to solid. The formulation of a mixture fraction approach provides a one-dimensional distribution of temperature (in a Z space). Generally, heat transfer can be considered as one-dimensional within the mixture fraction space near a stoichiometric surface. However, the heat transfer seems to be multidimensional at the flame front area near the burning surface. In this study, we aimed to develop a method to address the multidirectional energy transfer occurring in the flame tip region. It appears that further analysis and effort are needed to properly resolve this process using a tabulated chemistry approach. The second solution to be tested is to take into account heat transfer explicitly from calculations of a gas-phase energy equation.

As for the difference in predictions and measurements of the thermal structure of the flame (Figure 9), we emphasize that the source of such error is the same as for flame spread rate, i.e., incorrect resolution of multidimensional heat flux nearby the flame front. Combustion of polymers is a specific phenomenon produced by an interaction between processes in the gas phase and in solid material. Its modeling requires accurate resolution of each such process. If heat transfer is predicted incorrectly, the model of solid material would give the wrong combustible volatiles mass supply rate. Next, the flame shape, size, and position would be changed. And, again, this would modify heat flux from flame to solid material.

By improving the accuracy of heat flux predictions from the flame to solid materials in the flame tip zone, one can more accurately predict flame spread behavior (such as flame spread rate, temperatures, and species distribution).

## 4. Conclusions

In the current work we made an attempt to numerically predict polymer burning behavior by a mixture fraction approach. A model case of flame spread over solid fuel’s surface was chosen due to its widespread usage by the scientific community and detailed theoretical description available. Also, PMMA was chosen as the polymeric sample for the same reasons.

A modified expression of mixture fraction based on elemental mass fractions was formulated to take into account fuel mixtures containing oxygen elements.

An approach was proposed to take into account heat losses from the gas phase to a solid material by calculating counterflow diffusion flames with the flame positioned close to the fuel supply. The combination of these solutions was applied to restore temperature and species mass fractions from tabulated chemistry. A skeletal chemical mechanism for gas-phase combustion was used, consisting of 29 species and 33 reactions.

An analysis of numerical results of previous studies of flame spread over PMMA based on the one-step combustion reaction and calculation of a chemical source term at each time step demonstrated the monotonous distribution of mixture fraction in a flame region between fuel and oxidizer streams.

The comparison of the numerical results obtained with the previous predictions without chemistry tabulation on the flame tip shape showed reasonable agreement. However, the heat flux from the flame to the solid fuel was overpredicted, leading to higher values of flame spread rate than observed in the experiment and previous calculations. Nevertheless, preliminary results show a promising opportunity for a mixture fraction approach to resolve the combustion of polymers.

Many assumptions have been used in the current study to simplify the approach formulated, but we suggest that the main source of an error is the simplification of heat transfer formulation in the vicinity of the flame front. In this area, resolution of multidimensionality of heat transfer is a key factor for proper prediction of flame spread behavior. Thus, the proposed approach needs to be improved by taking this factor into consideration. The second option is to solve the gas-phase energy equation directly during calculations.

These problems are going to be resolved in the future work. As for now, the presented approach allowed the building of an appropriate shape of flame tip at the flame front in comparison with calculations without chemistry tabulation.

## Data Availability

The original contributions presented in this study are included in the article. Further inquiries can be directed to the corresponding author.
